# Mathematical Modeling and Physicochemical Characterization of Foam-Mat Drying of Acerola (*Malpighia emarginata*) Pulp

**DOI:** 10.3390/foods15030492

**Published:** 2026-02-01

**Authors:** Leandro Fagundes Mançano, Eliane Mauricio Furtado Martins, Fernanda Machado Baptestini, Gabriel Henrique Horta de Oliveira

**Affiliations:** 1 Centro Federal de Educação Tecnológica Celso Suckow da Fonseca, Campus Valença, Valença 27600-845, RJ, Brazil; leandro.mancano@cefet-rj.br; 2 Instituto Federal de Educação, Ciência e Tecnologia do Sudeste de Minas Gerais, Campus Rio Pomba, Rio Pomba 36180-000, MG, Brazil; eliane.martins@ifsudestemg.edu.br; 3 Department of Rural Engineering, Universidade Federal do Espírito Santo, Campus Alegre, Alegre 29500-000, ES, Brazil; fernanda.baptestini@ufes.br; 4 Instituto Federal de Educação, Ciência e Tecnologia do Sudeste de Minas Gerais, Campus Manhuaçu, Manhuaçu 36909-300, MG, Brazil

**Keywords:** dehydration, kinetics, fruit

## Abstract

This study aimed to evaluate the physical properties of foam produced with different additives and to mathematically model foam-mat drying at different temperatures. Foams were made with 500 g of acerola pulp and 4% additives (albumin, emustab, and neutral alloy), and density and stability data were obtained. With suitable density (0.14367 g cm^−3^) and coalescence (0.0000 mL) values, emustab provided the best acerola pulp foam. Drying was performed at temperatures of 50, 55, 60, 65, and 70 °C, using emustab, and different mathematical models were fitted for each temperature. The fresh pulp and the dried powder were analyzed for water activity, moisture content, ash, pH, total titratable acidity, soluble solids content, reducing sugars, vitamin C, total carotenoids, and instrumental color. The logarithmic model showed the best fit for all temperatures, with increasing k-values (between 0.0021 and 0.0059 s^−1^) and effective diffusion coefficient (between 2.570 × 10^−9^ m^2^ s^−1^ and 5.060 × 10^−9^ m^2^ s^−1^) with increasing temperature. Temperature directly impacts the effective moisture diffusion coefficient with an activation energy of 30.88 kJ mol^−1^. The physicochemical properties of the foams varied significantly with increasing drying temperature. Among all the temperatures tested, 60 °C was the most appropriate to reduce changes in nutritional composition.

## 1. Introduction

The acerola (*Malpighia emarginata* L.) is a tropical fruit from the Americas that is highly appreciated for its high content of vitamin C and as a source of B vitamins. It is widely consumed both in nature and in the form of derivative products, such as pulp [1,2]. However, since the pulp is a food with high water content, postharvest deterioration reactions occur, reducing its shelf life [3,4,5].

Cold processing can increase the shelf life of these types of products but can also increase their cost. Alternatively, the use of dehydration reduces the volume and mass of the product through water loss, decreasing transportation and storage costs [6]. Foam mat dehydration of heat-sensitive-liquid or semi-liquid foods, such as acerola, ensures reduced drying time and temperature, low cost, and obtaining a porous and easily rehydrated product compared to the conventional method [7,8]. The success of this method is linked with the correct use of foaming agents. Among these, albumin, emustab, and neutral alloy can be used.

Emustab is an emulsifier widely used in the food industry due to its low-cost, easy handling, and ability to incorporate air well without forming agglomerates. It is sold in paste form and contains compounds considered important for food technology, such as distilled fatty acid monoglycerides (surfactant/aerating agents), sorbitan monostearate (stabilizer), and polyoxyethylene sorbitan monostearate (surfactant). Most of these compounds are of lipid origin and are considered safe products [9].

Albumin is a high-biological-value protein derived from egg white, which has an excellent capacity for forming foams, gels, and emulsions. It provides hydrophobic interactions, intermolecular disulfide bonds, and ionic interactions that result in interfacial properties. These interactions also allow its use in the formation of foams and air-in-liquid emulsions, due to the ability of hydrophobic groups to reorient themselves to the gas phase and hydrophilic groups to the liquid phase after mechanical agitation [10].

Neutral alloy is an industrial product used as a thickener that contains sucrose, carboxymethylcellulose, and guar gum, compounds derived from carbohydrates, and is mainly used in the ice cream, bread, and confectionery industries. This product is sold in powder form, acting in the absorption and fixation of free water, with a consequent increase in viscosity, improving the texture and making the product creamier, which helps in the formation of a stable foam [11]. Also, it is a cheap and easy-to-use product.

Previous work reported the foam-mat drying of acerola pulp in particular situations [12,13,14,15]. Araújo et al. [12] evaluated the kinetics of the foam-mat drying of acerola pulp with different concentrations (2%, 4%, 6%, 8% and 10%) of albumin and emustab, indicating the best concentration (4%) and the best foaming agent (emustab). Matos et al. [13,14] studied the foam-mat drying of the mixed pulp of acerola and jambolan and its physical, bioactive, and physicochemical qualities. Paiva et al. [15] investigated the effect of the combination of albumin and gum Arabic additives, albumin and guar gum, albumin and gelatin, in the foam-mat drying of the blend of acerola, guava, and pitanga.

For the food industry, the knowledge of different foaming agents is of great importance to prevent quality loss and thus economic depreciation. The interactions provided by foaming agents directly impact drying kinetics. To study this phenomenon, mathematical models are used, along with the diffusion coefficient and activation energy [16].

As previous work analyzed albumin and emustab and indicated 4% concentration for stable foams but not for acerola pulp drying, the first objective of this study was to test neutral alloy as an alternative foam agent, and compare it with albumin and emustab, according to the density and coalescence of the foams. Secondly, the best foaming agent with acerola pulp would be used to conduct the drying experiments. In addition, we aimed to determine the physical–chemical characteristics of the fresh and dried pulp, to evaluate the drying kinetics of acerola pulp in a foam mat at different temperatures, and to fit mathematical models to describe this operation.

## 2. Materials and Methods

The preparation of the foam and the drying of the fresh acerola pulp were performed at the Beverages Technology Laboratory of the Federal Center for Technological Education Celso Suckow da Fonseca (Centro Federal de Educação Tecnológica Celso Suckow da Fonseca—CEFET-RJ), Valença Campus.

The material used in the research, such as the fruit, albumin, emustab, and neutral alloy, was acquired in the local market in Valença-RJ, Brazil, and later processed to obtain the pulp. Finally, the foam was obtained to perform the drying.

### 2.1. Sample Preparation

Initially, the fruits were selected, with deteriorated ones being discarded. Next, the fruits that were considered healthy were weighed and washed to remove coarse dirt and sanitized with a chlorine concentration of 100 ppm. Afterwards, the fruits were triturated in an industrial blender (JL Colombo manufacturer, Itajobi, Brazil, 19 L) with deionized water in the proportion 13 parts pulp to 4 parts water, according to the pre-tests, and then filtered in a domestic stainless-steel sieve with a diameter of 24 cm. The filtered pulps were heat-treated at 95 °C for one minute and kept frozen at −18 °C until further use.

#### 2.1.1. Foaming Agent

Three different foam-forming additives were tested at a fixed concentration of 4.0% (*w*/*w*): albumin, emustab, and neutral alloy.

#### 2.1.2. Preparation and Determination of Physical Properties of Foams

Foams were prepared and their physical properties were determined according to a methodology adapted from [12]. The methodology consists of submitting the pulp mixture (500 g) with each additive at 4.0% (*w*/*w*) to stirring in a Venâncio VBP 05 planetary mixer for 15 min. Pre-tests (coalescence and density) were made, using the previous work’s [12] method. During the initial 5 min, the minimum speed of the mixer (96 rpm) was used, and over the next 10 min, the speed was 214 rpm. The physical properties evaluated for each foam were density and stability (coalescence).

The densities of the foams were determined with the help of a 500 mL beaker, where the density was calculated by the ratio between the mass of foam and the volume occupied by the foam, expressed in g/cm^3^, according to Equation (1).

(1) $$\rho =\frac{{Mass}_{glassware+sample}-{Mass}_{empty\;glassware}}{Volume\;occupied\;by\;the\;sample}$$

The stability was determined with the help of a 1 mL beaker, by leaving it at rest for one hour and then measuring the height of the coalesced residue at the bottom of the glassware, using a ruler, with subsequent calculation of the volume (Equation (2)), expressed in milliliters (mL), the result obtained being inversely proportional to the foam stability.

(2) $$Coalesced\;volume=\pi r^{2}A$$

in which r is the radius of the beaker and A is the height of the coalesced liquid.

### 2.2. Study of the Drying Kinetics of Foam

As proposed by [12], after choosing the foaming agent, the study of the drying kinetics of the foam was performed in a BOD incubator (Limatec, Cruz das Almas, Brazil), model LT 20 UT with forced air circulation at temperatures of 50, 55, 60, 65, and 70 °C. For this, the freshly prepared foam was spread on circular aluminum trays (dimensions 35.5 cm in diameter and 1.5 cm in height). The foam layer filled the full tray height and was subjected to dehydration. In the first hour of drying, the samples were weighed on a analytical balance (KnWaagen, Cotia, Brazil), model KN 3200/2, every 20 min; in the following 2 h, every 30 min; and, from this point on, every hour, until the mass was constant in three consecutive weighings. The data from the weighings were used for the moisture ratio (MR) calculations, according to Equation (3).

(3) $$MR=\frac{M-M_{e}}{M_{0}-M_{e}}$$

in which M is the product moisture at time t, in % dry; M_e_ is the equilibrium moisture content, in % dry; and M_0_ is the initial moisture, in % dry, determined according to Section 2.2.1, Equation (5). The other moistures were obtained by calculating the loss in mass of the samples during the drying times, according to Equation (4).

Equilibrium moisture content was determined as stated in Section 2.2.1, after drying at each temperature.

(4) $${Moisture}_{dry\;foam}\;(\%)=\frac{P_{0}-P_{t}}{P_{e}}\times 100$$

in which P_0_ is the initial mass of water in the sample in foam form, in grams; P_t_ is the mass of water lost from the product at time t (time), the moment of weighing, in grams; and P_e_ is the mass of the dry sample at equilibrium, in grams.

#### 2.2.1. Moisture

The determination of moisture content followed the analytical standards of the Association of Official Analytical Chemists [17], consisting of weighing 5 g of the sample in a porcelain crucible previously heated to 105 ± 1 °C, cooling it in a desiccator to room temperature, and subsequently recording the mass. The crucible-plus-sample set was submitted to a oven (Nova Ética, Vargem Grande Paulista, Brazil), model 402-6DE, at 105 ± 1 °C for 24 h and then cooled in a desiccator to room temperature and weighed again. The result was expressed on a percentage (*w*/*w*) dry basis for pulp foam, according to Equation (5).

(5) $${Moisture}_{foam}\;(\%)=\frac{100\times M_{foam}}{R}$$

in which M_foam_ is the mass of water loss of the sample, in grams, and R is the mass of the dry residue of the sample after analysis.

#### 2.2.2. Mathematical Modeling

Once the MR data were calculated, they were subjected to nonlinear regression analysis at the 5% significance level to verify the fit of the Lewis, Page, and Henderson and Pabis mathematical models—Logarithmic, Modified Midilli, Diffusion Approximation, and Two-Term Exponential—to the experimental data, as described in [8,18].

(6) $$MR=e^{-kt}$$

(7) $$MR=e^{-kt^{n}}$$

(8) $$MR={a\;e}^{-kt}$$

(9) $$MR{=ae}^{-kt}+b$$

(10) $$MR{=e}^{-kt^{n}}+bt$$

(11) $$MR{=ae}^{-kt}+(1-a)e^{-kbt}$$

(12) $$MR{=ae}^{-kt}-\left(1-a\right)e^{-kat}$$

in which “t” is the drying time in seconds; “k” is the drying constant, s^−1^; “a”, “b”, and “n” are the empirical model constants, dimensionless; and “MR” is the moisture ratio, dimensionless, represented by Equation (3).

The choice of the best model was based on the coefficient of determination (R^2^), the standard deviation of the estimate (SDE), and the mean relative error (MRE); the calculation of the latter two was performed using Equations (13) and (14). The model that showed results less than 10% for the mean relative error, higher values for the coefficient of determination, and lower values of the standard deviation of the estimate at all temperatures was selected to describe the foam-mat-drying kinetics of acerola pulp [19].

(13) $$MRE=\frac{100}{n}\sum \frac{\left|Y-\hat{Y}\right|}{Y}$$

(14) $$SDE=\sqrt{\frac{\sum {\left(Y-\hat{Y}\right)}^{2}}{DF}}$$

In which MRE is the mean relative error, %; n is the number of observed data; Y is the observed value, % d.b.; Ŷ is the value estimated by the model, % d.b.; SDE is the standard deviation of the estimate, % d.b.; and DF is the degree of freedom of the model.

#### 2.2.3. Effective Diffusivity and Activation Energy

Considering that the moisture in the thin-layer drying process only flows in one direction, the expansion is negligible, the diffusion is constant, the initial moisture distribution is uniform, and the external resistance is negligible, Equation (15) can be applied [20].

(15) $$MR=\frac{8}{\pi^{2}}\;\sum_{n=0}^{\infty }\frac{1}{{\left(2n+1\right)}^{2}}exp\left(-\left(2n+1\right)\pi^{2}\frac{D_{eff}t}{4L^{2}}\right)$$

in which D_eff_ is the effective moisture diffusivity (m^2^ s^−1^), t (s) is drying time, n is a positive integer, and L is ½ material thickness (m).

Equation (16) is obtained by taking the natural logarithm of both sides. Plotting the experimental drying data as ln (MR) against drying time allows for the determination of the diffusion coefficient, with D_eff_ representing the slope of the straight-line regression equation (Equation (17)).

(16) $$\ln MR=\ln\frac{8}{\pi^{2}}-\pi^{2}\frac{D_{eff}t}{4L^{2}}$$

(17) $$Slope=\pi^{2}\frac{D_{eff}}{4L^{2}}$$

The Arrhenius equation (Equation (18)), which illustrates how the effective diffusion coefficient depends on the drying air temperature, was used to compute the activation energy.

(18) $$D_{eff}=D_{0}exp\left(-\frac{E_{a}}{RT}\right)$$

in which D_o_ is the pre-exponential factor (m^2^ s^−1^), E_a_ is the activation energy (J mol^−1^), R is the universal gas constant (8.314 J mol^−1^ K^−1^), and T is the temperature (K).

### 2.3. Physicochemical Characterization of Fresh Acerola Pulp and Acerola Powders

#### 2.3.1. Moisture Content, Water Activity, and Ash

The determination of moisture content followed the analytical standard of the Adolfo Lutz Institute [21], standard 012/IV, consisting of weighing 5 g of the sample in a porcelain crucible previously heated to 105 ± 1 °C, cooling it in a desiccator to room temperature, and subsequently recording the mass. The crucible-and-sample set was subjected to an oven (Nova Ética, Vargem Grande Paulista, Brazil), model 402-6DE, at 105 ± 1 °C for 24 h and then cooled in a desiccator to room temperature and weighed again. The results were expressed on a percentage wet basis (Equation (19)):

(19) $$X\;=\;\frac{100\times \;M}{P}$$

in which X is the moisture content, % w.b.; M is the water mass lost, g; and P is the sample weight, g.

Water activity was determined in an Aqualab Lite apparatus (Addium Meter Group, São José dos Campos, Brazil) at 25 °C.

The ash content was determined according to the analytical procedures of the Adolfo Lutz Institute [21], standard 018/IV, by weighing 5 g of the sample in a porcelain crucible, previously heated in a muffle furnace to 550 °C, cooled in a desiccator to room temperature, and weighed. Before performing the analysis, the sample was subjected to pre-carbonization on a heating plate (IonLab, Araucária, Brazil, model XMTD-204) at a temperature of 100 °C. After this procedure, the sample was sent to the muffle furnace (Digimec, São João Clímaco, Brazil, model SH-1) at 550 °C, until complete elimination of carbon for 5 h. After this procedure, the crucibles were removed from the muffle furnace, transferred to the desiccator until they reached room temperature, and weighed again before being sent back to the muffle furnace. This heating and cooling procedure was repeated until the ash was white or slightly grayish in color and had a constant mass. Results were presented in ash percentage (Equation (20)):

(20) $$Ash\;=\;\frac{100\times \;N}{P}$$

in which Ash is the ash percentage, %, and N is the ash weight, g.

#### 2.3.2. Analysis of pH, Total Titratable Acidity, Soluble Solids Content, Reducing Sugars, Vitamin C, and Total Carotenoids

The pH analysis was conducted following standard 017/IV of the Adolfo Lutz Institute [21]. After correct calibration of the pH meter (MS Tecnopon, Piracicaba, Brazil, model mPA-210), the electrode was removed from the buffer solution and washed with deionized water. In the case of the liquid fresh pulp sample, there was no prior preparation, taking the direct reading of 50 mL of the sample in a 100 mL beaker. However, for the powdered pulp samples, 5 g of the sample was weighed, 50 mL of distilled water was added, and the sample was homogenized in a domestic blender (Mondial, Conceição do Jacuípe, Brazil, Ultra model L-25) for 10 s at the lowest speed, representing a ratio of 1:10 (powder/distilled water). Then, the electrode was placed inside the beaker with the sample, we waited for the reading to become constant, and then the value was recorded.

The total titratable acidity analysis was determined by the potentiometric volumetry method, standards 311/IV and 312/IV, as recommended by the Adolfo Lutz Institute [21]. Initially, the pH meter (MS Tecnopon, Piracicaba, Brazil, model mPA-210) was calibrated with buffer solutions of pH 8, 7, and 4. Then, 5 g of the sample was weighed into a 250 mL beaker, diluted with 100 mL of water, homogenized, and, finally, the electrode was inserted into the sample solution under homogenization on a magnetic stirrer (IonLab, Araucária, Brazil, model XMTD-204). Using a 25 mL burette, the sample was titrated with the standardized 0.1 M sodium hydroxide solution to a pH range (8.2–8.4). Results were expressed as citric acid (g 100 g^−1^), Equation (21):

(21) $$TTA\;=\;\frac{V_{NaOH}\times f\times M_{NaOH}\times MW}{10\times P\times n}$$

in which TTA is the total titratable acidity, g 100 g^−1^; V_NaOH_ is the spent volume of sodium hydroxide 0.1 M, mL; f is the correction factor of the sodium hydroxide solution, dimensionless; M_NaOH_ is the molarity of the sodium hydroxide solution, mol L^−1^; MW is the molecular weight of the corresponding acid, g mol^−1^; and n is the number of ionizable hydrogens, dimensionless.

The soluble solids content of the fresh pulp sample was determined by the refractometry method, using a digital refractometer (Nova Instruments, Piracicaba, Brazil, model NOVA DR-500), standard 315/IV, according to the method described by the Adolfo Lutz Institute [21]. The soluble solids content of the powdered product followed the method described by Soares et al. [22], which consisted of diluting the sample in a ratio of 1:10 (powder/distilled water) with the aid of a domestic blender (Mondial, Conceição do Jacuípe, Brazil, Ultra model L-25) for 10 s at minimum speed and subsequent measurement in the portable digital refractometer, previously calibrated with distilled water. The result was expressed in °Brix at 25 °C, with corrections being made to the value obtained from the analysis of the diluted powdered sample, considering the proportion used.

To determine the reducing sugar content, the analytical method proposed by the Adolfo Lutz Institute, standard 038/IV, was used [21]. This consisted of initially weighing 2 g of the sample in a 100 mL beaker. The sample was then transferred to a 100 mL volumetric flask with the subsequent addition of deionized water until the volume was complete, and the solution was homogenized under manual stirring. Using dry filter paper and a glass funnel, the sample was filtered into a 250 mL Erlenmeyer flask, and the filtrate was transferred to a 50 mL burette. Each of the standardized Fehling solutions A and B (10 mL each) was added to a 250 mL Erlenmeyer flask using 10 mL pipettes, followed by the addition of 40 mL of water. This solution was heated on a hotplate with magnetic stirring (IonLab, Araucária, Brazil, model XMTD-204) until boiling. After boiling began, three drops of methylene blue (1%, *w*/*v*) were added to the Fehling solution, along with the sample solution in the burette as the titrant. The titration was carried out with the sample still boiling under mechanical stirring until the solution changed from blue to colorless (a red Cu_2_O residue appeared at the bottom of the flask). The reducing sugar content was calculated with Equation (22):

(22) $$RS\;=\;\frac{100\times DV\times T}{V\times P}$$

in which RS is the reducing sugar content, g_glicose_ 100 g^−1^; DV is the volume in which the samples were diluted, mL; T is the title of the Fehling solution, dimensionless; and V is the used volume in titration, mL.

Vitamin C analysis was performed using the method recommended by the Adolfo Lutz Institute, standard 364/IV [21]. This analysis consisted of weighing 1 g of the sample, transferring it to a 250 mL Erlenmeyer flask with 50 mL of water, and adding 10 mL of a 20% (*m*/*v*) sulfuric acid solution with a 10 mL pipette. The sample solution was then homogenized and filtered using filter paper into another 250 mL Erlenmeyer flask, washing the filter with water and with 10 mL of 20% (*m*/*v*) sulfuric acid. Once the filtrate was obtained, 1 mL of a 10% (*m*/*v*) potassium iodide solution and 1 mL of a 1% (*m*/*v*) starch solution were added to it. This solution was then titrated with a standardized 0.002 M potassium iodate solution for fresh samples and 0.02 M for dry samples, as per pre-tests, using a 25 mL burette until a blue color was obtained. Equation (23) was used to calculate the vitamin C content.

(23) $$VC=\frac{100\times V_{{KIO}_{3}}\times F}{P}$$

in which VC is the vitamin C content, mg 100 g^−1^; V_KIO3_ is the volume of potassium iodate used in the titration, mL; and F is the value 8.806 or 0.8806, respectively, for a KIO_3_ of 0.02 M or 0.002 M, dimensionless.

The carotenoid content was determined by spectrophotometric analysis, according to the methodology of the Adolfo Lutz Institute, standard 124/IV [21]. Approximately 70 mg of the sample was weighed into a 50 mL beaker, which was transferred to a 500 mL volumetric flask, completing the volume with deionized water. Using a volumetric pipette, a 20 mL aliquot of the sample was collected and transferred to a separatory funnel. 80 mL of acetone was added using a 100 mL graduated cylinder, and the solution was stirred for 5 min. Then, 60 mL of petroleum ether was added using a 100 mL graduated cylinder, followed by deionized water to aid the transfer of the pigment to the ether phase. After phase separation, the lower phase was discarded, and the solution was washed three more times with approximately 150 mL of water. After washing, the resulting solution was collected in a 250 mL Erlenmeyer flask containing anhydrous sodium sulfate to remove water droplets and transferred to a 100 mL volumetric flask, which was then made up to volume with petroleum ether. An aliquot was collected using a 5 mL pipette and transferred to a quartz cuvette. The absorbance was read on a digital spectrophotometer (Agilent Technologies, Santa Clara, CA, USA, model Cary 60 UV/Vis) at a wavelength of 450 nm, using petroleum ether as a blank at zero reading. The result was obtained using Equation (24) [23].

(24) $$TC=\frac{Abs\times V_{aliquot}\times 10^{4}}{A_{1\mathrm{cm}}^{1\%}\times P}$$

in which TC is the total carotenoid content, µg 100 g^−1^; Abs is the absorbance of the solution at a wavelength of 450 nm, dimensionless; V_aliquot_ is the volume of the aliquot collected after diluting the sample, mL; and $A_{1\mathrm{cm}}^{1\%}$ is the absorptivity coefficient of carotenoids in petroleum ether, whose value is equal to 2592.

#### 2.3.3. Color Analysis

The instrumental color coordinates (CIE L*a*b* system) were determined using a portable colorimeter (DeltaColor, São Leopoldo, Brazil, DeltaVista model d8), which displays the values of the coordinates a*, b*, L*, chroma or saturation (C*), and hue angle (h*). L* indicates lightness, assuming a value of 0 (zero) for absolute black and 100 for total white (the higher the values, the lighter the sample—the lower the values, the darker the sample); C* represents saturation; h* the hue angle; and a* and b* are the chromatic coordinates in which the higher the value of a, the redder the sample, and the lower the values of this coordinate, the greener the sample (+a indicates red and −a indicates green); higher values of b indicate a more yellow sample, while the lower the values of this coordinate, the bluer the sample (+b indicates yellow and −b indicates blue) [24]. From the values of the parameters L*, a*, and b*, the browning index (BI) was calculated using Equations (25) and (26), with the result expressed in a dimensionless form [25]. This parameter represents the degree of brown color present in a dry sample.

(25) $$BI=100\left(\frac{Y-0.31}{0.17}\right)$$

(26) $$Y=\frac{a^{*}+1.75L^{*}}{5.645L^{*}+a^{*}-3.012b^{*}}$$

### 2.4. Statistical Analysis

The experimental design used in this project was entirely randomized, with three replicates in duplicate, and the statistical analyses of the experimental data were performed using STATISTICA 14.0 software.

The data concerning the physical properties of the foams produced with different additives, at a concentration of 4%, were submitted to Analysis of Variance (ANOVA), at a 5% significance level, and compared by Tukey’s test, also at a 5% significance level. The additive that provided the lowest density and highest stability was used for the drying kinetics study.

Physicochemical parameters that showed a significant difference in relation to the drying temperatures were subjected to linear regression analysis, also at a significant level of 5%. The process of choosing the most suitable drying temperature to obtain acerola pulp powder dried in a foam bed was based on the optimization of the equations of the physicochemical parameters that showed a significant difference as a function of increasing temperature. Each equation was manually entered into the GeoGebra Calculator program and, using this software, individually optimized to obtain its ideal maximum or minimum. Then, with the data, these were subjected to the calculation of the average to find the ideal midpoint between the equations.

## 3. Results and Discussion

### 3.1. Choice of the Foaming Agent

The results of the physical properties of the foams, density and coalescence, are shown in Table 1.

It was observed that the foam made with albumin showed the highest density (0.83858 g/cm^3^) and largest coalesced volume (492.3333 mL) compared to the other additives (*p* < 0.05), demonstrating that this additive was not efficient for the elaboration of acerola pulp foam. Neutral alloy showed intermediate density (0.55595 g/cm^3^) and coalesced volume (26.5867 mL) values, and emustab, lower density (0.14367 g/cm^3^) and coalesced (0.0000 mL) values (*p* < 0.05).

Since the stability of a foam can be influenced by the chemical nature of the raw materials, soluble solids content, and type of foaming agent [26], it can be inferred that the interaction of acerola pulp components with albumin and neutral alloy was not effective in making a stable foam (Table 1). From these results, we can conclude that comparing foaming agents with the same concentration does not ensure comparable foam structure or stability, even though previous work indicates a specific concentration for stable foams [12]. Thus, each foaming agent should be optimized individually, or comparisons should be made at similar functional foam properties (e.g., density, viscosity, or stability), rather than at the same mass fraction.

However, Mançano et al. [27], in their studies on the foam-mat-drying kinetics of Haden mango pulp, found that there was a good foam formation when using albumin (0.41317 g/cm^3^ density and 0.0133 mL coalesced volume). It was noticed that there was no statistical difference in density and coalesced volume, at the 5% level, with results found for the mango pulp foam prepared with emustab (0.42753 g/cm^3^ of density and 0.0933 mL of coalesced volume).

In their constitution phases, pulps are formed by solid particles dispersed in aqueous medium; thus, the concentration, chemical composition, and distribution of the particles that make up the dispersed phase are determinants that dictate the behavior of the pulp of each fruit and are necessary to evaluate different agents for each pulp [28,29].

Thus, the results found in the present study corroborate previous work [28,29], demonstrating that different fruit pulps can behave in different ways when using the same additive in foam making.

A stable foam has around 0.1 to 0.6 g/cm^3^ of density [30], contributing, along with stability, to a quick drying process, since the lower the density of the foam, due to its greater porosity, the easier and faster the water diffusion process through the foam during dehydration.

Although the neutral alloy showed density within the range indicated by [30], it showed a coalesced volume of around 26.5867 mL, which is undesirable. Therefore, albumin and neutral alloy were excluded from the rest of the experiment, because their foam proved to be more unstable and could not withstand the drying conditions. Emustab, on the other hand, due to its excellent density and coalescence values, was chosen to perform the kinetics of foam-mat drying.

Such result was also observed by Araújo et al. [12] when evaluating the drying kinetics of acerola pulp in a foam mat, using albumin and emustab; as well as also observed by Feitosa et al. [31], in their studies on the drying and characterization of myrtle pulp, using neutral alloy and emustab as foamers.

### 3.2. Study of the Drying Kinetics of Foam

According to Chicco et al. [32] and Souza et al. [19], the coefficient of determination (R^2^) is a parameter widely used for the selection of mathematical models to represent the behavior of experimentally observed phenomena, but it is not a good criterion when used alone since it is more suitable for linear models. Therefore, the standard deviation of the estimate (SDE) and mean relative error (MRE) are commonly applied when the objective of the study is to analyze the fit quality of non-linear models. The MRE values reflect the deviation of the experimental data from those estimated by the model, with results less than 10% being the recommended results in model selection [19].

According to Table 2, it was observed that the model that presented good fits to the experimental data, with R^2^ values higher than 0.99, MRE values lower than 10%, and lower SDE results (lower than 0.036) for all temperatures was the Logarithmic model, which was chosen to represent the behavior of moisture over time.

This situation in which MRE was lower only for the Logarithmic model, as cited by Keneni et al. [33], may be related to the periods of a decreasing rate in which, at lower temperatures, these drying stages may extend over time, making the moisture loss behavior linear and thus making modeling difficult. As none of the models studied predicts this phenomenon, this behavior may have reflected in the results of the present study, generating MRE values for most models at some temperatures higher than 10%, which contradicts the criteria recommended [19].

As shown in Table 2, for the Logarithmic model, it is possible to observe that the drying coefficient “k” increased with increasing temperature, while the constant “a” and “b” did not show a trend as a function of increasing drying temperature. As “k” represents the constant of the drying rate, the values observed in the present experiment corroborate what is expected for this parameter, since, when the temperature is raised, there is an increase in the speed of water loss from the product to the environment.

Experimental data of persimmon pulp drying in the foam mat was fitted [34], and the authors also found the Logarithmic model to be the best, as well as previous study of mango pulp drying in foam mat [35]. In both studies, an increase in the values of the parameter “k” with increasing temperature was also noticed, while parameters “a” and “b” did not show a trend as a function of drying temperature.

**Figure 1:**
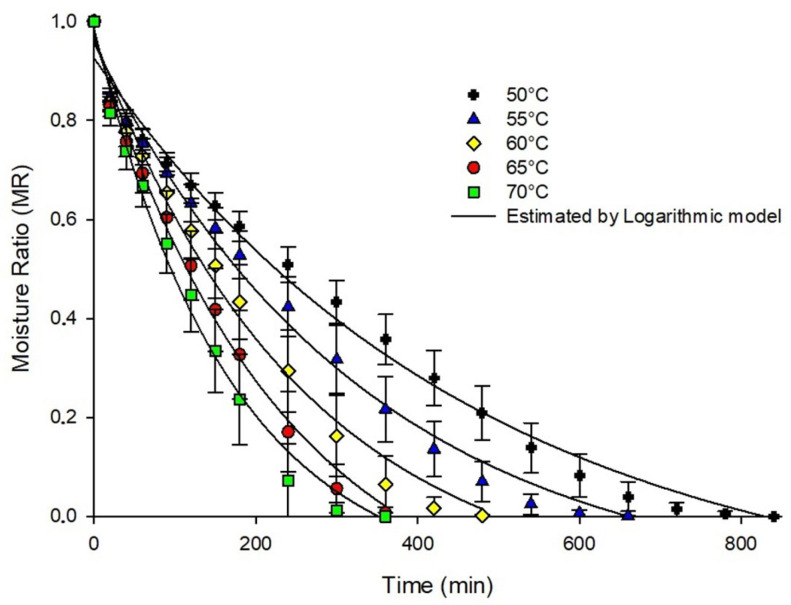
Moisture content (RU) as a function of time of acerola pulp on a foam mat at temperatures of 50, 55, 60, 65, and 70 °C.

It was observed that the drying time decreased when increasing the temperature from 50 to 70 °C (Figure 1). The acerola pulp foam reached the equilibrium moisture faster at 70 °C (300 min) while at 50 °C, this time was longer (840 min). This behavior proves that raising the drying temperature has a great influence on the moisture loss rate of the material studied.

During dehydration, a temperature differential is created between the food and the drying air, forming a vapor pressure gradient that promotes water removal [6,36,37]; an increase in temperature raises this differential as well as reduces the relative humidity of the air.

In addition, rapid moisture loss is observed in the early stages of drying, and as the final period of the process approaches, the rate of moisture loss reduces for all temperatures. Generally, during the dehydration process, the rate of moisture loss is higher in the early stages, due to high moisture contents (presence of free water) in the sample; while in the later stages, there is a drop in the rate of water removal, due to the presence of water strongly bound to the constituents of the food [33].

This trend in moisture behavior was also observed by Li et al. [38] when evaluating the effect of gum arabic concentrations on foam properties, drying kinetics, and physicochemical properties of melon foam-mat drying; by El-Salam et al. [39] when performing a mathematical modeling and quality evaluation of papaya pulp dehydrated in foam mat; and by Matos et al. [13] when studying the drying kinetics in foam mat of mixed pulp of jambolan and acerola.

The values of D_eff_ were 2.570 × 10^−9^; 3.234 × 10^−9^; 4.149 × 10^−9^; 4.427 × 10^−9^; 5.060 × 10^−9^ m^2^ s^−1^_,_ respectively, for an air temperature of 50 °C, 55 °C, 60 °C, 65 °C and 70 °C. This trend is expected, since effective moisture diffusivity rises because of increased mass transfer brought on by greater drying air temperatures. According to some earlier research, the values obtained fall within the appropriate range for a variety of products [40]. Arrhenius equation calculates the activation energy and the linear relationship between ln (D_eff_) and the drying temperature inverse (1/T) is highly correlated with an R value of 0.9604. Using the regression equation, the activation energy estimated value for the acerola pulp foam drying process is 30.88 kJ mol^−1^, within the general range of 14.42–43.26 kJ mol^−1^, previously reported for the drying of fruits and vegetables [41]. Furthermore, the range 12–40 kJ/mol indicates that the physical process occurring during the drying of the acerola pulp foam is described by the moisture loss with temperature and time [20]. It is important to indicate that, because of the model’s limitation (assumption of constant foam thickness, constant structural of the foam during drying), the calculated diffusion coefficient and activation energy may present error in its calculations. Future research should address this trend. However, it is important to notice the behavior of these parameters.

### 3.3. Physicochemical Characterization of Fresh Acerola Pulp and Acerola Powders

#### 3.3.1. Moisture Content, Water Activity and Ash

A difference was observed between the samples dried at different temperatures and the fresh pulp sample (*p* < 0.05), with a loss in moisture content, an increase in ash content, and a decrease in water activity (Table 3).

According to the Brazilian Food Composition Table [42], the moisture and ash contents of the fresh acerola pulp observed in this study are adequate, since the results found in this study are close to those expressed in the aforementioned table: values around 90.5% and 0.400%, respectively, for moisture and ash contents.

The moisture content (Table 3) decreased as the temperature increased, ranging from 21.338 to 16.894% w.b. However, when subjected to temperatures of 60 and 65 °C, there was no significant difference (*p* ≥ 0.05), nor at temperatures of 65 and 70 °C (*p* ≥ 0.05), possibly due to the process and/or climatic conditions that may influence the result of this parameter. This behavior in reducing the moisture content was also observed in the physical–chemical characterization of soursop powder obtained by foam-bed drying [43].

Thus, the temperature used in the process directly influences the moisture content of the final product, since higher temperatures promote increased heat transfer, reducing drying time (Table 3). As a result, there will be greater evaporation of water from the sample, resulting in products with lower moisture contents [36,37]. Also, the drying equipment used has forced air circulation, but not air renovation. This trend permitted the foams to reach equilibrium with the inside air conditions at each temperature, leading to a higher final moisture content than predicted.

The ash content ranged from 3.714 to 4.342% (Table 3), with 65 °C (3.714%) having the lowest ash content (*p* < 0.05). However, the variation between samples was small and can be attributed to sample variability, such as foam homogeneity. Furthermore, the ash content values presented in Table 1, for all drying temperatures, are higher than the value obtained from fresh acerola pulp (0.377%). Similar data was found in [44], in which the authors analyzed the influence of foam-bed drying on the characteristics of uvaia pulp. This increase may be correlated with the concentration of pulp components after drying, which consequently increases the ash content; as well as with the addition of emustab (composed of distilled fatty acid monoglycerides, sorbitan monostearate, and polyoxyethylene sorbitan monostearate) to pulp samples, which may contribute to an increase in the content of this parameter [9].

Water activity (a_w_) is the parameter used to measure the amount of water available in each food and, thus, it regulates and limits the biological activities of microorganisms, and chemical and enzymatic reactions. It is very important data for dictating the storage and marketing conditions of the product [10]. Regarding this parameter (Table 3), the values identified for the fresh sample were 0.985, showing a difference between the powdered samples dried in a foam bed (*p* < 0.05), thus being considered a food with a high degree of perishability. However, the samples dried at temperatures of 50, 55, 60, 65, and 70 °C did not show a significant difference (*p* ≥ 0.05), presenting a_w_ results lower than or equal to 0.410. Previous work also found that water activity did not show a significant difference in their studies when using saponin and horse chestnut extracts as foaming agents in the foam-bed drying of pomegranate juice [45].

Since the powdered samples obtained through drying at different temperatures did not show a significant difference, with values between 0.326 and 0.410 (*p* ≥ 0.05), these are in accordance with previous work [38,46]. A water activity below 0.6 is ideal for a food to be considered microbiologically stable during storage [38], since microbial activities are suppressed below these values. However, other research indicated that the range between 0.35 and 0.5 is adequate to avoid the loss of desirable properties of dehydrated foods due to oxidation [46].

#### 3.3.2. Analysis of pH, Total Titratable Acidity, Soluble Solids Content, Reducing Sugars, Vitamin C and Total Carotenoids

There were differences between the dried samples and the fresh pulp sample for all parameters, except for pH, as shown in Table 4. This trend indicates a concentration effect on acidity, soluble solids, reducing sugars, and vitamin C after the removal of most of the aqueous portions of the acerola pulp. The alterations of these parameters should not be interpreted as intrinsic physicochemical or nutritional improvements, but rather as consequences of moisture reduction. According to Gonzaga et al. [47], the drying process removes most of the water from the food, which leads to the concentration of the components present in this sample. Consequently, the total soluble solids, reducing sugars, and other parameters increased. However, total carotenoids decreased with increasing drying temperature. A reduction in carotenoid content represents the most physically and chemically meaningful processing effect, as it is associated with thermal and oxidative degradation of these heat-sensitive compounds.

According to Normative Instruction No. 37 of 1 October 2018, which establishes the identity and quality standards for fruit pulp, the minimum requirements for pH, total acidity, total soluble solids, and ascorbic acid for acerola processing are 2.8; 0.8 g 100 g^−1^ in citric acid; 5.5 °Brix; and 800 mg 100 g^−1^, respectively [48]. Therefore, the pulp used for drying complied with current legislation (Table 4).

The reducing sugar content of acerola pulp can vary within a range of 1.82% and 8.92% depending on the degree of ripeness [49,50]. In this study, sugar values of 5.290% of the fresh pulp were found, which are within the literature.

Regarding the total carotenoid content, the reference value is approximately 247 µg 100 g^−1^ [42], and the value found in this study for fresh acerola pulp is 252.00 µg 100 g^−1^, being appropriate.

As shown in Table 4, the pH parameter ranged from 3.626 to 3.686, demonstrating stability across the drying process temperatures. For all samples analyzed, the results were below 4.5, indicating that the acerola powder remained acidic, not requiring the addition of preservatives to prevent the development of microorganisms.

The total titratable acidity values ranged from 5.272 to 4.689 g 100 g^−1^ (Table 4), presenting a decreasing trend as the temperature increased. This observation may be related to the possible oxidation of organic acids during the drying process [47]. A similar result was observed previously [50] for the drying of a byproduct generated in the pearl pineapple-processing industry. It should be noted that acerola contains organic acids such as malic, citric, and tartaric acid, with citric acid being the most predominant [3,4,5].

The total soluble solids varied in values from 27.666 to 30.333 °Brix; however, there was no significant difference (*p* ≥ 0.05) between samples dried at different temperatures, also found by Kandasamy et al. [51]. It should be noted that the low values identified for total soluble solids may be related to the phenomenon of solids that were previously soluble becoming insoluble due to the drying process. The total soluble solids represents all solids dissolved in water, such as sugars, minerals, proteins, and acids. They are extremely important in quality control, as they are a parameter used for consumer acceptance, as they estimate the sweetness of fruits and, therefore, are responsible for flavor [43].

Regarding the reducing sugar content, the drying temperature influenced this parameter, as there was a tendency for the reducing sugar content to increase with increasing temperature (*p* < 0.05). An increase in temperature tends to increase the hydrolysis of oligosaccharides and polysaccharides present in the fruit to monosaccharides (reducing sugars) [52]. This parameter varied in concentration between 31.603 and 36.472% (Table 4).

The carbohydrates found in fruits are classified as mono-, oligo-, and polysaccharides. Monosaccharides are considered non-hydrolysable simple sugars, while oligosaccharides and polysaccharides are formed by monosaccharide molecules linked by glycosidic bonds. In this context, reducing sugars are those with free carbonyl and ketone groups that are oxidized in the presence of oxidizing agents in alkaline solutions (monosaccharides), while non-reducing sugars are those without these free groups (oligosaccharides and polysaccharides) [10].

According to Table 4, a trend toward increasing vitamin C content was observed as the temperature increased. This observation suggests a possible correlation between the pulp’s exposure time and drying temperature. As the temperature increased, there was an improvement in heat and mass transfer, which in turn reduced the process duration (Table 3). Therefore, given that higher temperatures resulted in samples with lower moisture content, this may have contributed to an increase in the concentration of solutes, including vitamin C. Similar observations were noted in [45]. However, it should be noted that vitamin C, also called ascorbic acid, is one of the vitamins most sensitive to heat, exposure to oxygen, moisture, and light, and, therefore, can be partially or completely degraded by the drying process [47]. The carotenoid content (Table 4) was highest at 50 °C (188.481 µg 100 g^−1^) (*p* < 0.05), while at other temperatures, there were considerable losses of this parameter, although there was no significant difference between the samples at 55, 60, 65, and 70 °C, with a range from 118.724 to 58.713 µg 100 g^−1^ (*p* ≥ 0.05).

In general, in contrast to vitamin C content, carotenoid content decreased as the temperature increased. This observation can be explained by the fact that carotenoids, also known as vitamin A precursors, are susceptible to oxidation and thermal degradation due to their unsaturated chemical structure [39]. Thus, as the exposure of carotenoids to air at high temperatures increases, degradation reactions may increase, thus causing their loss, as verified in the present work.

#### 3.3.3. Color Analysis

Table 5 shows the difference between the dried samples and the fresh pulp in terms of the L* color coordinate, ranging from 55.556 to 62.1530, indicating an increase in luminosity at initial drying temperatures, with a subsequent reduction in this parameter at final temperatures.

When comparing the luminosity coordinate result for the fresh pulp (55.583) with the results at initial temperatures, this increase in the parameter may be related to the addition of the emustab additive and the stirring process to obtain a stable foam. Incorporating air into the product may have favored the color change, making the sample lighter. This observation was also obtained by Cól et al. [46] when using albumin and emustab to obtain a foam and subsequent drying.

An increase in the results of color coordinates a* and b* (Table 5) can be observed after the application of drying. This indicates that, although the b* parameter did not present a significant difference between the dehydrated samples (*p* ≥ 0.05), this phenomenon may be correlated with the occurrence of non-enzymatic browning reactions such as Maillard and caramelization reactions [6,10]. These reactions occur under the influence of the heat of drying, causing a change in the color of the dried foods, and may also have caused a browning effect observed in the results of the L* parameter as a function of the increase in temperature. These findings were also reported by [39], who observed an increase in the darkening of dehydrated samples with increasing drying temperature when drying papaya pulp in a foam bed.

Human vision utilizes a complex combination of electromagnetic wave frequencies and intensities within a visible field to produce perceived colors. These can be represented analytically using the parameters hue angle (h*), lightness (L*), and chroma (C*). These three attributes, together, can quantify human color perception within a spectrum [53].

An increase in hue angle (h*) was observed at initial temperatures, compared to fresh acerola pulp, with a decrease in these values as the drying temperature increased (Table 5). This behavior was similar to that of the color coordinate L*. This occurrence may be related to the foaming process that employed the emustab additive and may have increased the hue angle results of the fresh acerola pulp at initial drying temperatures. It is related to non-enzymatic browning reactions, such as Maillard and caramelization reactions, during the process, which may have contributed to the reduction in the hue angle results at final drying temperatures [6,43]. However, although the hue angle behavior decreased as the temperature increased, its value ranged between 0° and 90° (red and yellow, respectively), regardless of whether samples were fresh or dried. It should be noted that the h* coordinate (hue angle) represents the dominant color or hue and ranges across 0°, 90°, 180°, and 270°, corresponding to the colors red, yellow, green, and blue, respectively [24].

When comparing the chroma results (Table 5) of the fresh acerola pulp (14.07°) with the dehydrated powder products, there was an increase in C* (*p* < 0.05), indicating that temperature influenced this parameter. Since chroma or chromaticity represents the saturation, intensity, or purity of the color in relation to gray [24], it can be inferred that the fresh pulp had an opaquer appearance, while the dehydrated pulp had a more vibrant appearance in terms of color. However, regarding drying, there was no significant difference as the temperature increased (*p* ≥ 0.05), which demonstrates color stability.

The phenomena observed in this study regarding hue angle and chroma were also similarly reported by Dehghannya et al. [54] in their study on the lemon-juice foam-bed drying process. The authors also found that increasing the process temperature can lead to darkening reactions, which leads to a decrease in the value of h*; however, they also noticed that the chroma value did not change when the temperature increased from 50 to 70 °C.

**Figure 2:**
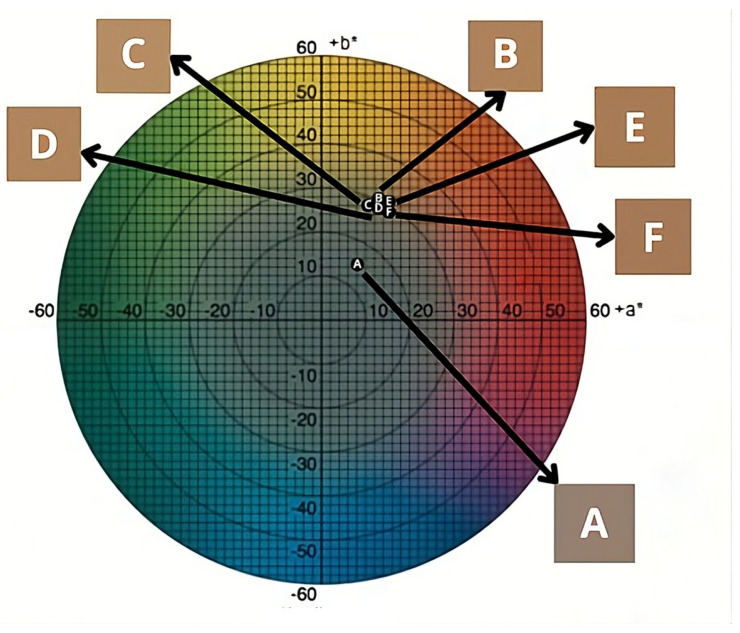
Color representation of each sample object in the chromaticity diagram.

Figure 2 shows the a* and b* chromaticity diagram of the raw acerola pulp (A) and the powders dried in a foam bed at temperatures of 50 (B), 55 (C), 60 (D), 65 (E), and 70 °C (F), with the respective color representations for each point expressed in the Cartesian coordinates of the diagram, taking into account the a* and b* values in Table 5.

Figure 2 shows the color difference between sample A (fresh acerola pulp) and the other pulp samples dried in a foam bed at different drying temperatures. However, when comparing the dried samples, no visible difference was observed between the samples through color analysis, possibly because there was no difference between the samples for the b* and C* parameters (*p* ≥ 0.05).

Based on visual perceptions, the browning index (Table 6) was calculated considering the results for the L*, a*, and b parameters. The browning index (BI) is an important parameter in the analysis of dehydrated products, as it is used to quantify the degree of browning in foods after drying, indicating whether non-enzymatic browning reactions occurred in the product [25,55].

A temperature of 55 °C resulted in less browning compared to temperatures of 50, 65, and 70 °C, while a temperature of 60 °C resulted in intermediate browning (*p* < 0.05). These results suggest that the samples dehydrated at 50 °C, exposed to the drying temperature for a longer period (840 min), as well as the samples dried at 65 and 70 °C, exposed to higher drying temperatures, may have undergone more intense Maillard reactions. These observations corroborate what is presented in Table 5, since, according to the results of the L* coordinate, a tendency towards a reduction in this parameter was observed, indicating darkening.

As shown in Table 7, it was possible to represent the behavior of the physicochemical parameters that presented significant differences at the 5% level using the Tukey test as a function of the drying temperature using quadratic models (moisture content, vitamin C, total carotenoids) and cubic models (ash, total titratable acidity, reducing sugars, L*, a*, and h* coordinates).

It was possible to obtain statistically significant models for all physicochemical parameters studied, as well as a significant fit for all coefficients of these models obtained (*p* < 0.05) with R^2^ greater than 0.81. On the other hand, for the total titratable acidity parameter, it was not possible to successfully obtain a model with all statistically significant coefficients (*p* ≥ 0.05) (Table 7).

Although the model obtained to represent the behavior of total titratable acidity as a function of temperature presented a statistically insignificant coefficient, this model proved to be statistically significant with an R^2^ above 0.90. This means that the variation in the acidity variable can be adequately explained by the temperature variable.

To select the temperature that had the least influence on the physicochemical characteristics, taking into account lower losses in vitamin C, reducing sugars, total titratable acidity, and carotenoids, as well as lower moisture contents and smaller changes in the color coordinates L*, a*, and h*, which showed significant differences, the optimization process was performed based on the equations presented in Table 7. After the optimization procedure, the optimal temperature was 59.5794 °C; therefore, the temperature chosen was 60 °C.

## 4. Conclusions

After evaluating the physical characteristics of the foams, it was found that those prepared with albumin and neutral alloy showed instability; hence, these were not recommended for use in foam-mat drying. On the other hand, the emustab additive allowed the obtainment of a stable foam, according to the recommendations in the literature, and this was chosen for the drying kinetics. Future work should consider optimizing each foaming agent individually, or using a specific foam property (e.g., density, viscosity, or stability), rather than using a specific mass fraction of the foaming agent.

By investigating the effect of temperature during the drying process, it was possible to observe how the physical–chemical properties of the fruit changed, i.e., ash, moisture, vitamin C, total carotenoids, reducing sugars, total titratable acidity and the color coordinates L*, a*, and h*. A reduction in carotenoid content is associated with thermal and oxidative degradation of these heat-sensitive compounds, whilst the other parameters are related to a concentration effect due to moisture removal after drying.

Among the mathematical models analyzed, the logarithmic model was the one that presented the best fit, since it showed the best results in the statistical coefficients for all temperatures.

The temperature that least influenced the physicochemical characteristics of the dried powder was 60 °C. Thus, this study can help food industries, as the powder obtained from drying at 60 °C has the potential for the development of new dairy and non-dairy products, such as whipped cream, snacks, yogurts, cookies, ice cream, breads, and nectars.

## Figures and Tables

**Table 1:** Physical properties of foams.

Additives	Density (g/cm^3^)	Coalescence (mL)
Albumin	0.83858 ± 0.0220 ^a^	492.3333 ± 0.5792 ^a^
Neutral Alloy	0.55595 ± 0.0012 ^b^	26.5867 ± 0.3562 ^b^
Emustab	0.14367 ± 0.0331 ^c^	0.0000 ± 0.000 ^c^

Means followed by the same letter do not differ in the column by Tukey’s test at 5% probability.

**Table 2:** Fitted parameters of the empirical models for temperatures of 50, 55, 60, 65, and 70 °C.

Model	Temperature (°C)	Coefficients				R^2^	MRE (%)	SDE (% d.b.)
		a	n	k	b			
Two-Term Exponential	50	1.5000	---	0.0040	---	0.9750	11.2733	0.0562
	55	1.5976	---	0.0053	---	0.9830	11.2792	0.0475
	60	1.6564	---	0.0071	---	0.9830	6.9340	0.0480
	65	1.6540	---	0.0087	---	0.9834	6.2375	0.0423
	70	1.6813	---	0.0104	---	0.9859	5.4117	0.0419
Lewis	50	---	---	0.0033	---	0.9730	12.2284	0.0529
	55	---	---	0.0042	---	0.9786	13.8277	0.0463
	60	---	---	0.0054	---	0.9763	8.1774	0.0454
	65	---	---	0.0066	---	0.9764	7.5100	0.0397
	70	---	---	0.0078	---	0.9784	5.8112	0.0392
Diffusion Approach	50	1.0605	---	0.0028	−0.1932	0.9824	9.1879	0.0540
	55	1.0000	---	0.0042	1.0000	0.9786	13.8277	0.0507
	60	1.0000	---	0.0054	1.0000	0.9802	8.2580	0.0523
	65	1.0000	---	0.0066	1.0000	0.9764	7.5098	0.0458
	70	1.0000	---	0.0078	1.0000	0.9784	5.8115	0.0464
Logarithmic	50	1.1143	---	0.0021	−0.1883	0.9902	7.8635	0.0360
	55	1.1104	---	0.0029	−0.1546	0.9919	9.5636	0.0335
	60	1.1280	---	0.0039	−0.1581	0.9903	5.6683	0.0349
	65	1.2020	---	0.0043	−0.2325	0.9934	4.4103	0.0294
	70	1.1241	---	0.0059	−0.1376	0.9908	4.1328	0.0323
Modified Midilli	50	---	0.2027	0.0821	−0.0011	0.9995	134.0286	0.3839
	55	---	0.2796	0.0554	−0.0014	0.9997	154.4380	0.3966
	60	---	0.5663	0.0226	−0.0012	0.9963	49.5096	0.2296
	65	---	0.9146	0.0079	−0.0004	0.9923	14.4232	0.0703
	70	---	1.0583	0.0052	−0.0001	0.9899	4.7994	0.0387
Page	50	---	1.0418	0.0026	---	0.9735	11.7987	0.0570
	55	---	1.1126	0.0022	---	0.9815	43.1378	0.1139
	60	---	1.1636	0.0023	---	0.9819	7.0712	0.0520
	65	---	1.1600	0.0029	---	0.9823	6.4321	0.0461
	70	---	1.1859	0.0031	---	0.9854	5.7742	0.0460
Henderson and Pabis	50	0.9640		0.0032	---	0.9751	12.3334	0.0486
	55	0.9902		0.0042	---	0.9787	14.0096	0.0473
	60	1.0033		0.0054	---	0.9963	8.2107	0.0486
	65	1.0066		0.0066	---	0.9765	7.6048	0.0431
	70	1.0145		0.0079	---	0.9899	5.8839	0.0437

**Table 3:** Average values and standard deviations of moisture content, ash and water activity of the acerola pulp in natura and the acerola powders obtained by drying in a foam bed as well as the drying time at temperatures of 50, 55, 60, 65 and 70 °C.

	Drying Time (min)	Moisture Content (%w.b.)	Ash (%)	Water Activity
Pulp	-	91.95 ± 0.02 ^a^	0.37 ± 0.11 ^c^	0.98 ± 0.00 ^a^
50 °C	840 ^d^	21.33 ± 0.37 ^b^	4.31 ± 0.05 ^a^	0.41 ± 0.02 ^b^
55 °C	660 ^c^	18.95 ± 0.25 ^c^	4.342 ± 0.16 ^a^	0.36 ± 0.01 ^b^
60 °C	480 ^b^	17.84 ± 0.32 ^d^	4.28 ± 0.17 ^a^	0.36 ± 0.05 ^b^
65 °C	360 ^a^	17.54 ± 0.18 ^de^	3.71 ± 0.06 ^b^	0.35 ± 0.02 ^b^
70 °C	300 ^a^	16.89 ± 0.19 ^e^	4.08 ± 0.16 ^ab^	0.32 ± 0.03 ^b^

Means followed by the same letter do not differ in the column by Tukey’s test at 5% probability.

**Table 4:** Mean values and standard deviations of the parameters pH, total titratable acidity (TTA), soluble solids content (SSC), reducing sugars (RS), vitamin C (VC), and total carotenoids (TC) of the acerola pulp in natura and of the acerola powders obtained by drying in a foam bed.

	pH	TTA (g 100 g^−1^)	SSC (°Brix)	RS (%)	VC (mg 100 g^−1^)	TC (µg 100 g^−1^)
Pulp	3.58 ± 0.03 ^a^	0.84 ± 0.29 ^a^	7.40 ± 0.16 ^a^	5.29 ± 0.07 ^a^	873.15 ± 1.92 ^a^	252.00 ± 4.96 ^a^
50 °C	3.68 ± 0.02 ^a^	5.27 ± 3.86 ^c^	29.33 ± 2.05 ^b^	32.44 ± 2.37 ^bc^	4013.82 ± 31.70 ^b^	188.48 ± 3.20 ^a^
55 °C	3.63 ± 0.01 ^a^	5.09 ± 2.81 ^bc^	27.66 ± 1.24 ^b^	31.60 ± 1.64 ^b^	5429.15 ± 29.48 ^c^	118.72 ± 9.53 ^b^
60 °C	3.66 ± 0.03 ^a^	5.00 ± 2.72 ^bc^	30.33 ± 2.05 ^b^	32.66 ± 0.89 ^bc^	6399.30 ± 36.44 ^cd^	82.83 ± 1.48 ^b^
65 °C	3.63 ± 0.03 ^a^	4.68 ± 1.78 ^b^	29.66 ± 0.47 ^b^	36.47 ± 0.54 ^c^	6964.80 ± 22.03 ^d^	68.10 ± 3.20 ^b^
70 °C	3.62 ± 0.03 ^a^	4.78 ± 1.58 ^bc^	29.00 ± 0.81 ^b^	34.10 ± 0.59 ^bc^	6953.49 ± 10.06 ^d^	58.71 ± 2.97 ^b^

Means followed by the same letter do not differ in the column by Tukey’s test at 5% probability.

**Table 5:** The mean values and standard deviations of the color coordinate parameters of the fresh acerola pulp and acerola powders obtained by drying in a foam bed at temperatures of 50, 55, 60, 65, and 70 °C.

	L*	a*	b*	h*	C*
Pulp	55.58 ± 0.56 ^ab^	6.28 ± 0.58 ^a^	12.57 ± 0.29 ^a^	63.46 ± 2.53 ^a^	14.07 ± 0.21 ^a^
50 °C	58.16 ± 1.63 ^ab^	11.02 ± 0.26 ^c^	28.89 ± 1.73 ^b^	69.04 ± 1.41 ^bc^	30.94 ± 1.57 ^b^
55 °C	62.15 ± 0.63 ^c^	9.01 ± 0.55 ^b^	27.800 ± 1.06 ^b^	72.05 ± 0.54 ^c^	29.22 ± 1.17 ^b^
60 °C	59.14 ± 0.92 ^bc^	11.26 ± 0.65 ^c^	28.10 ± 0.16 ^b^	68.15 ± 1.13 ^abc^	30.28 ± 0.31 ^b^
65 °C	56.16 ± 1.02 ^ab^	12.62 ± 0.48 ^c^	28.50 ± 0.88 ^b^	66.07 ± 1.35 ^ab^	31.18 ± 0.68 ^b^
70 °C	55.55 ± 1.21 ^a^	12.51 ± 0.53 ^c^	26.12 ± 1.69 ^b^	64.36 ± 0.54 ^ab^	28.96 ± 1.75 ^b^

Means followed by the same letter do not differ in the column by Tukey’s test at 5% probability.

**Table 6:** Acerola powder browning index as a function of drying temperature.

	BI
50 °C	79.89 ± 2.72 ^b^
55 °C	67.94 ± 0.61 ^a^
60 °C	76.20 ± 0.40 ^ab^
65 °C	84.48 ± 2.28 ^b^
70 °C	77.78 ± 0.49 ^b^

Means followed by the same letter do not differ in the column by Tukey’s test at 5% probability.

**Table 7:** Regression equations as a function of drying temperature (T) and their respective coefficients of determination (R^2^), mean relative errors (P), and errors of estimate (SDE).

Parameter	Regression Equations	R^2^	MRE (%)	SDE
Moisture content (X)	$X=0.0122^{*}T^{2}-1.6715^{*}T+74.2269^{*}\;$	0.9777	1.0503	0.3763
Ash	$Ash={-0.0027}^{*}T^{2}+0.295^{*}T-3.6958^{*}$	0.9980	3.7922	0.3567
Total titratable acidity	$TTA=-0.0296^{*}T+6.7451^{*}$	0.9836	1.2865	0.1023
Reducing sugar content	$RS=0.0122^{*}T^{2}-1.2357^{*}T+63.081^{*}$	0.9791	3.2955	2.2848
Vitamin C content	$VC=-9.3083^{*}T^{2}+1265.2979^{*}T-35990.4010^{*}$	0.9996	0.3169	1.0906
Total carotenoid content	$TC=0.4054^{*}T^{2}-54.853^{*}T+1914.7668^{*}$	0.9927	3.9449	0.2020
Lightness	$L^{*}=0.0108^{*}T^{2}+1.2481^{*}T-30.872^{*}$	0.9829	2.7215	3.4313
Coordinate a*	$a^{*}=-0.009^{*}T^{2}-41.89^{*}T+840.3328^{*}$	0.9489	8.4598	1.8192
Hue angle	$h^{*}=0.004^{*}T^{2}-0.8504^{*}T+104.26^{*}$	0.9813	1.6814	2.6125

The asterisk (*) sign represents a coefficient with a significant parameter at a 5% level.

## Data Availability

The raw data supporting the conclusions of this article will be made available by the authors on request.
